# Climate crisis, health equity, and democratic governance: the need to act together

**DOI:** 10.1057/s41271-019-00209-x

**Published:** 2020-01-21

**Authors:** Nancy Krieger

**Affiliations:** Department of Social and Behavioral Sciences (Kresge 717), Harvard T.H. Chan School of Public Health, 677 Huntington Avenue, Boston, MA 02115 USA

**Keywords:** Civic engagement, Climate crisis, Democracy, Gerrymandering, Health equity, Voter suppression

## Abstract

On Friday, 20 September 2019, over 4 million people worldwide participated in the youth-led Global Climate Strike. Emphasizing the dire impacts of the climate crisis on people’s health, planetary health, and health equity, participants called for politicians and those with power to listen to the scientists and to the evidence. But who are these politicians and what is the evidence regarding to whom they listen? In the United States (US), critical research documents how the public’s will is being subverted—and people and planetary health are being harmed—via changes to the ‘rules of the game’ that affect democratic governance. Health professionals, organizations, and institutions should encourage civic engagement—for themselves, their staff, members, and study participants—regarding: voter registration; being counted in the 2020 Census; countering partisan gerrymandering; and helping to build strong coalitions addressing profound links between climate change, health equity, and democratic governance.

On Friday, 20 September 2019, over 4 million people worldwide participated in the youth-led Global Climate Strike [1]. There were more than 1000 such strikes in the US alone [1]. I rescheduled the large required graduate-level public health course I teach on Friday mornings so that the students and I could participate in the strike rally that took place at Boston City Hall Plaza, which drew over 7000 participants [2]. Tellingly, the day before, a new study reported that in the past 50 years the bird population in North America had declined by over 30 percent [3]; Rachel Carson’s decision to title her 1962 opus *Silent Spring* never felt more prescient [4].

Across the globe, the strike organizers, speeches, banners, and the bright hand-lettered signs waving everywhere emphasized the dire impacts of the climate crisis on people’s health, planetary health, and health equity [1, 2]. All called for politicians and those with power to listen to the scientists and to the evidence—to guide fruitful urgent action to avert climate disaster and to secure a right to a future for generations to come. The same demand was forcefully issued by Greta Thunberg—the 16-year-old Swedish climate activist whose solo “school strike for climate” outside the Swedish parliament in 2015 has morphed into a global movement—in her powerful address to the United Nations on Monday, September 23, 2019 [5].

Notably, the public health and medical communities have provided critical evidence about the myriad adverse—and highly inequitable—health impacts of climate change [6–8]. But is this the only evidence that requires our scientific attention? And is it a sufficient guide to action?

I would suggest no. Instead, we in the health professions—whether in public health or health care providers—must also pay attention to and take action regarding another body of evidence little discussed in the public health or medical literature: the critical research documenting how the public’s will is being subverted—and people and planetary health are being harmed—via changes to the ‘rules of the game’ that affect democratic governance [9–15]. These rules matter, because they structure who become the politicians and people in power, and thus whom they listen to and on behalf of whose interests they take action [9–15]. To strengthen the political impact of evidence on the health impacts of the climate crisis, it is essential to develop an informed and cohesive approach to addressing the conjoined political and biophysical threats to the people’s health and planetary health that is as every bit as integrated as how our bodies, like the bodies of all living organisms, daily embody the societal and ecological conditions in which we live [16].

In the case of the United States (US), an extremely wealthy and powerful political minority has been spending lavishly to change the rules of the game to undermine the democratic majority [10–15], thereby making it increasingly difficult to protect people’s health and have a thriving democracy. Key tactics include [10–15]:

1. political gerrymandering, using highly sophisticated computerized techniques, which enables politicians to pick voters, rather than have voters elect politicians;
2. voter suppression, justified via false allegations of voter fraud, including imposition of highly burdensome voting identification (ID) requirements, plus cutting the locations and hours of polling places; and
3. undermining, including underfunding the US Census, whose population counts are fundamental to determining voting districts and also the allocation of resources for the more than 300 federally funded programs whose program-specific funding formulas take into account population size.

Some of these US political tactics to subvert good governance, moreover, are being picked up by other countries, for example, a new and unprecedented proposal in the UK to ramp up identification (ID) requirements for voting, despite a similar absence of evidence of voter fraud [17].

In all cases, these political stratagems are linked by the desire to weaken or eliminate regulations that crimp the ever-growing concentration of power and wealth, orchestrated and funded especially but not only by those with a financial stake in the fossil fuel and petrochemical industries and energy markets [10–14]. In the US, an example par excellence is provided by the economically and politically powerful Koch Industries, a private corporation whose profits began with oil refineries, but which has long since extended its reach into producing and trading innumerable physical and financial commodities [11–14]. Currently, the personal wealth of its two key owners (Charles and David Koch) is estimated to be $120 billion, more than that of either Bill Gates (founder of Microsoft) or Jeff Bezos (CEO of Amazon) [14, p. 5]. In the ‘Kochland’ view, the sole function of government is “to protect private property and do little else” [14, p. 5]. Only recently have major works been published that carefully detail how the Koch Industries’ early work to avoid, subvert, and change environmental regulations, dating back to the 1970s, had, by the 1990s, transformed into far larger efforts to change rules governing elections and legislation [11–14]. While the initial rationale was to avoid dealing with fines and court cases for breaking rules, for example, by weakening regulations to make previously illegal practices permissible, the expanded efforts have sought to remake government more generally. Extending well beyond traditional political lobbying, the Koch brothers have funded major think tanks and academic centers, with an objective of changing the content and contours of governance from the state to federal level, so as to bring to fruition their narrow vision of government, as solely guarantor of private property and markets [11–14].

Why has changing the rules of governance, and not just particular laws, say, about environmental regulations, become so central? Because the political and economic objectives of Koch Industries and kindred wealthy elite fly in the face of public opinion and voters’ preferences [11–15]. Their policy aims, in a nutshell, are to [11–15]:

- slash taxation on the wealthy and curb governmental financial regulations that restrict private wealth accumulation;
- minimize (or even eliminate) government regulation of land use by—and pollution produced by—the petrochemical, fossil fuel, and other industries (including financial) from which this wealth derives; and
- cut funding of government-sponsored health and social services that protect people’s health and improve well-being.

Each and every one of these aims is counter to the kinds of policies necessary for improving population health and reducing health inequities [6–9, 16].

Although many of these elite strategies to subvert democratic governance are far from unique to the US [11–15, 17], in the US these efforts are profoundly racialized, rooted in deep histories of settler-colonialism, slavery, and previously legalized and still culturally embedded white supremacy [10, 12]. Current efforts to limit the franchise and suppress voter turnout continue to be overly and often overtly directed against people of color, especially in low-income areas, in part to undercut these communities’ potential support for more inclusive government policies for economic well-being and environmental justice, and in part to get white ‘buy-in’ for the larger political agenda of protecting and entrenching existing systems of privilege [10].

The intensifying and grievous harms to the body politic—affecting political representation, power, and who literally counts—inexorably harm the people’s health and planetary health. At a time of unprecedented federal assaults on US policies to address climate change and to protect the environment and the public’s health, the links between democratic governance, the climate crisis, and health equity become clearer every day [6–8, 18–20].

Yet, typically, advocates for healthy democracies, for climate justice, and for population health and health equity do their work separately, each focused on their own issues [6, 11]. While specialization and expertise are necessary, so too is an awareness of the integuments that hold us together—biologically, socially, ecologically, and terrestrially [6–8, 16, 18–20]. The proverbial ‘parts’ cannot contribute to the well-being of the ‘whole’ if there is no clear articulation of this ‘whole’, let alone the links between the parts and the whole. The Green New Deal—in its national and global forms—provides one such integrated vision [20], which valorizes societal, ecological, community, and individual well-being, premised in respect for human dignity, human rights, equitable economies, and the bounded reality of living together on our one phenomenal and threatened planet.

In Table 1, I delineate some steps that US public health and medical institutions, agencies, and organizations could take (but have rarely taken) to foster links between work supporting democratic governance, tackling the climate crisis, and health equity. Articulating these action-oriented practical steps matters because having a positive vision of what to do is as necessary as naming the harms that need to be prevented [18, 20]. To date, the public health and medical literature remains scarce to non-existent on the health-related and climate-related harms of political gerrymandering and voter suppression [21], and, to a lesser degree, the census undercount [22]. We can do better: to advance not only the public health and medical scientific evidence in relation to these issues of subverted democratic governance, but also to apply the existing social science evidence on these powerful drivers of human and planetary health harms and health inequities. For the health of current and future generations, of people and of the other species with whom our lives are interdependent, we can afford no less.

**Table 1:** Practical action steps for civic engagement and democratic governance to promote equitable and integrated policies to address the climate crisis and build health equity

Action steps	Resources
(1) Promote voter registration *Action* Provide information about voter registration to staff, patients, members, and study participants *Who should take action* public health and medical agencies, facilities, schools, professional associations, non-profit organizations, and scientific studies	A non-partisan guide on how to register to vote in one’s state is provided by the League of Women Voters; see: https://www.vote411.org/
(2) Promote an accurate Census 2020 count *Action* Provide information about the importance of the census count to staff, patients, members, and study participants *Who should take action* public health and medical agencies, facilities, schools, professional associations, non-profit organizations, and scientific studies	The US Census Bureau has a website explaining why it is important to participate in the decennial Census (see: https://2020census.gov/en/what-is-2020-census.html) and it has established partnerships in communities throughout the US to encourage participation for an accurate count (see: https://census.gov/programs-surveys/decennial-census/2020-census/complete_count.html). Related information about the value of participating in the US Census American Community Survey is at: https://www.census.gov/programs-surveys/acs/
(3) Promote awareness of efforts to prevent voter suppression and partisan gerrymandering *Action* Educate staff, patients, members, and study participants about efforts in their state and nationally to prevent voter suppression and to curtail partisan gerrymandering *Who should take action* public health and medical agencies, facilities, schools, professional associations, non-profit organizations, and scientific studies	Information on the history of gerrymandering is available from the Encyclopedia Britannica at: https://www.britannica.com/topic/gerrymandering; the political geometry of gerrymandering is explained at: https://mggg.org/ Information on the history and current realities of voter suppression in the US are available at: https://people.howstuffworks.com/voter-suppression.htm and https://www.lwv.org/voting-rights/fighting-voter-suppression
(4) Promote awareness of the impacts of the climate crisis on population health and health inequities *Action* Educate staff, patients, members, and study participants about the impacts of the climate crisis—not just on population health overall, but also on how it exacerbates existing health inequities and creates new ones *Who should take action* public health and medical agencies, facilities, schools, professional associations, non-profit organizations, and scientific studies	A useful compendium of resources on “Climate Change, Health, and Equity” has been compiled by the American Public Health Association, at: https://www.apha.org/topics-and-issues/climate-change/guide A short new video (September 2019) featuring youth leaders in health and climate activism from around the world is available from the United Nations Development Programme (UNDP) at: https://youtu.be/NpXG3b8_rls
(5) Join in coalitions that foster links between health equity, social justice, climate justice, such as the Green New Deal, and also good governance *Action* Educate staff and members about the importance of working in coalition not only with the myriad groups working explicitly on issues of health equity, but also with groups working to advance social justice, climate justice, and good governance—and to foster, with evidence and with resources, the links between all of these groups *Who should take action* public health and medical agencies, facilities, schools, professional associations, and non-profit organizations	Green New Deal (GND) https://www.greennewdealforall.org/supporting-groups/ https://www.sunrisemovement.org/gnd/ https://www.dataforprogress.org/green-new-deal/ &video https://theintercept.com/2019/04/17/green-new-deal-short-film-alexandria-ocasio-cortez/ Global GND https://www.c40.org/other/the-global-green-new-deal Good governance (non-partisan groups) League of Women Voters: https://www.lwv.org/ Fair Fight: https://fairfight.com/
